# The best of 2005 in echocardiography back from EuroEcho 9 – Florence, Italy

**DOI:** 10.1186/1476-7120-4-11

**Published:** 2006-02-15

**Authors:** Rosa Sicari

**Affiliations:** 1 Institute of Clinical Physiology, CNR Pisa, Italy

## Abstract

The ninth edition of the congress of the European Association of Echocardiography (EAE) (former working group of Echocardiography) held in Florence has just finished with a great success of participant attendance (2.842) and abstract submissions. Hot topics at EuroEcho 9 were: 1-live 3-dimensional echocardiography and surgical decision making; in pediatric cardiology; in resynchronization therapy 2- stress echocardiography beyond wall motion: from valve diseases to contractility to coronary flow reserve to diastolic function; 3- pulmonary cardiogenic interstitial thickening recognized by ultrasonic lung comets; 4- the "proven clinical inefficacy" of the many technologies sold as breakthrough: color kinesis, tissue characterization, strain rate, tissue Doppler, applied to stress echocardiography.

The Meeting of the European Association of Echocardiography (EuroEcho9) that took place in Florence from December 6th to 10th, has just finished achieving the record of abstract submissions and participant attendance (2.842). The interest on ultrasound technologies is very high and is still increasing over time in relation to its widespread employment, often outside the cardiological community. This is a brief report on what went on for those who could not attend the meeting or could not follow all the sessions.

## Elections

During the opening ceremony, the president Alan Fraser announced the result of the recent elections for a new member in the Board. The winner was Prof. Albert Varga from Szeged, Hungary. He has an outstanding curriculum characterized by some key publications on safety of stress echocardiography and evaluating new protocols for the detection of myocardial viability. His election is very welcome inside the Board since he represents an independent and highly appreciated voice of the new European Union countries. For the first time the Association employed a completely electronic voting system (different from the previous one that was conducted via e-mail). The electoral system employed by the Association allows that any echocardiographist from any associated country member (the membership of countries to the European Association does not correspond to the 25 members of the European Union but is much larger), can submit his/her resume to the Board. The candidatures do not go through a selection system unless more than five candidates apply for the same position, as stated in the bylaws of the Association. In that particular case the Board will select the best profiles that would fit for that position. The electronic vote posed some technical problems (many physicians found the access a little bit too complicated) but the time allowed for voting was long enough to overcome these limitations. This is an open and transparent way to have access to the Board independent of country of origin and power of the echo working groups. In fact, if it is true that the two societies with the largest number of members in Europe (Italy and Poland) do have three members each in the Board, it is also true that much smaller working groups were able to elect their representatives such as Romania or Hungary. In the near future a new election for two critical positions, secretary of the Association and President will be held. It would be advisable that the endorsement of the national working groups and/or societies for candidatures were optional and not mandatory: the need for the support of national societies might exclude those who do not lead an active political life in their home societies but nonetheless can represent large sections of the European scientific community on the basis of their curriculum. This problem might be overcome with the institution of a co-membership fee that would make all the national working groups constituent bodies of EAE.

## The making of imaging guidelines

During the ceremony it was announced with great emphasis the imminent submission to the European Heart Journal of a position paper on the future of non-invasive imaging modalities in cardiovascular diagnosis. The joint statement will be signed by Alan Fraser and Petros Nihoyannopoulos on behalf of the EAE, Peter Buser and Jürg Schwitter on behalf of the Working Group on Cardiovascular Magnetic Resonance, Jeroen Bax and Juhani Knuuti on behalf of the Working Group on Nuclear Cardiology; Radiology Associations were invited to join among the authors in order to have a widespread acceptance, of general criteria to be applied every time an imaging technique is employed in the clinical practice. The first draft made available to the members of the Board of EAE raised many questions with an overwhelming feeling of not having taken any position regarding imaging modalities. The general view stems from the idea that all the techniques were created equal and can be used in the same clinical setting provided that they are non-invasive and performed by cardiologists. In fact, "no competition among modalities" is the key phrase of the paper. Too little to provide clearer indications to a cardiological community that is pressured between marketing forces giving birth to the latest high-tech indispensable machine and a scientific community unable to speak up sound and loud on the many conceivable limitations and risks of imaging techniques. The aim of the paper should be to create the intellectual and political framework to move from the culture of waste to the culture of responsibility and safety, in which the only possible competition of imaging modalities should be run on the basis of their cost-effectiveness, biological risks, safety, with the ultimate scope of providing key clinical and additive information to the patient [1].Nonetheless, authors promised to amend the manuscript on the basis of the criticism that was made. Those who have the responsibility of setting the pace (writing statements and/or guidelines) should take into consideration not only their personal view but also patient's needs and the impact of potentially wrong or neutral recommendations that will influence clinical practice [2]. I am sure that all these aspects will become the standard practice in the clinical evaluation of an imaging modality.

## Hot topics in scientific sessions

**Figure 1:**
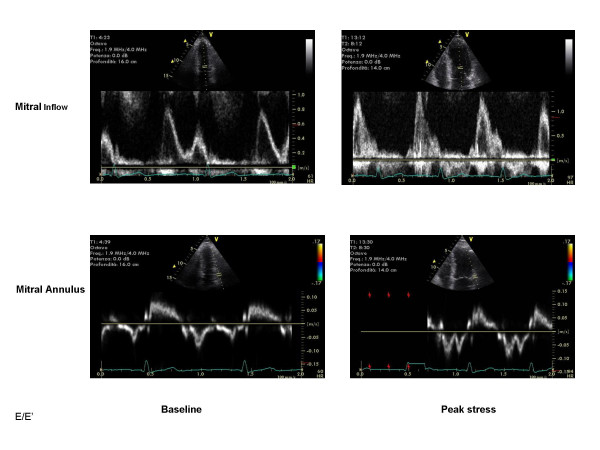
Mitral inflow and annulus velocity pattern in 70-year-old man with exertional dyspnea. Left, Mitral flow pattern at baseline is normal. Right, Mitral flow pattern changed during exercise of 100 W, which caused increase in E/mitral annulus early diastolic velocity (E1) ratio (from 9 at rest to 12 with exercise).

**Figure 2:**
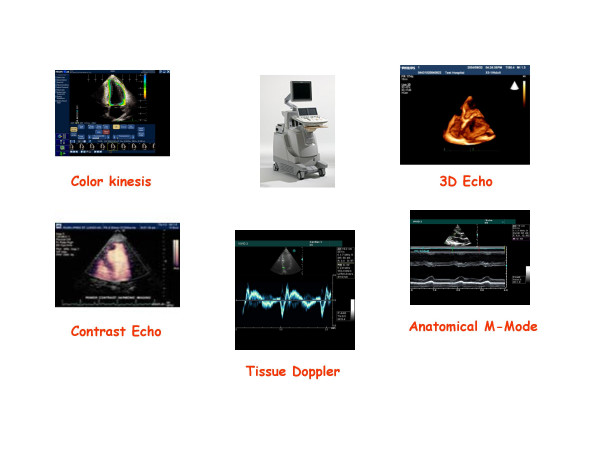
The garden variety of new technologies present on commercially available machines.

**Figure 3:**
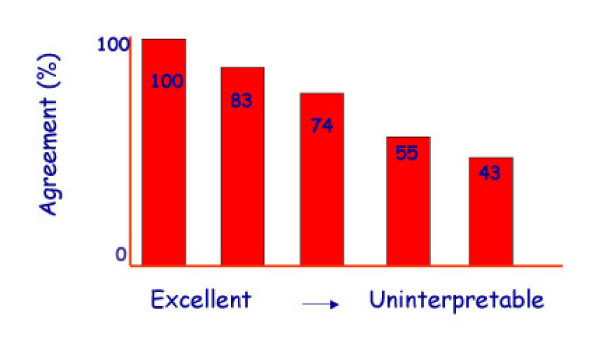
Stress echocardiography is a highly subjective, operator-dependent technique but different factors might affect its accuracy. The presence of good to uninterpretable images reduces significantly the agreement among expert readers. Good acoustic windows make stress echo interpretable in most cases.

**Figure 4:**
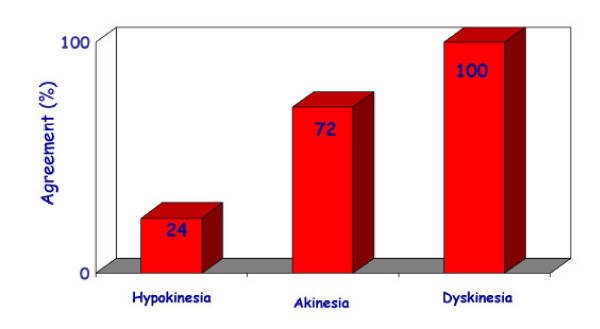
In the same line as the previous figure, hypokinesia represents the most inaccuarate type of dyssynergy to be interpreted. On the opposite end the agreement on dyskinesia reaches a 100% among expert readers.

**Figure 5:**
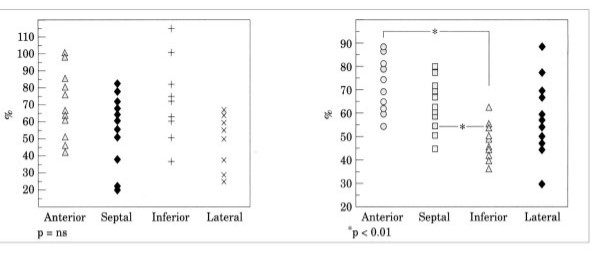
In the figure is reported % systolic thickening at rest and after dobutamine challenge. It is important to remind that not all segments were created equal and an induced dyssynergy at peak stress in the basal segment of the inferior wall is a normal stress echocardiographic response.

**Figure 6:**
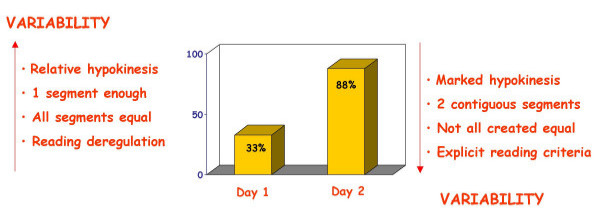
In order to reduce stress echo reading variability, few simple rules can be employed: disregard relative hypokinesia, test positivity only in the presence of at least two dyssynergic segments of the same vascular territory, consider the physiologic differences of segments during stress (basal inferior), adopt laboratory explicit rules on stress echo protocols and response.

**Figure 7:**
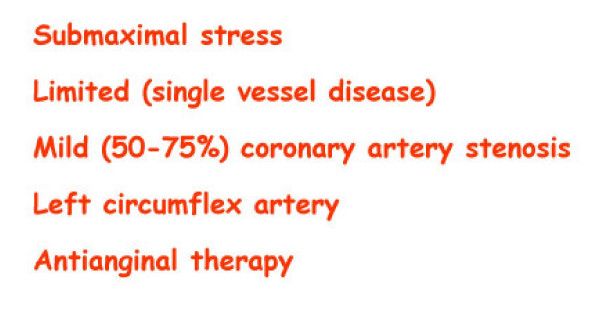
Reasons for a submaximal stress echo test.

**Figure 8:**
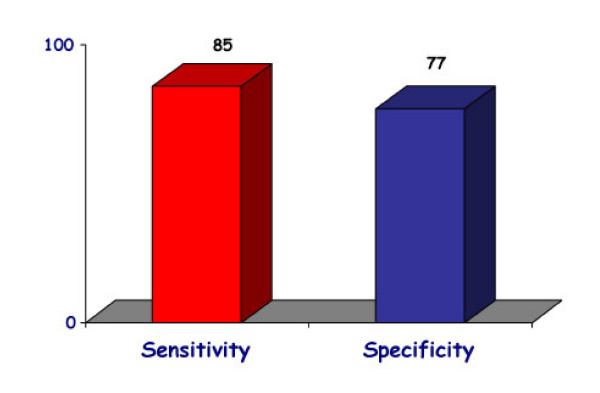
The overall sensitivity and specificity of exercise stress echocardiography in a meta-analysis.

**Figure 9:**
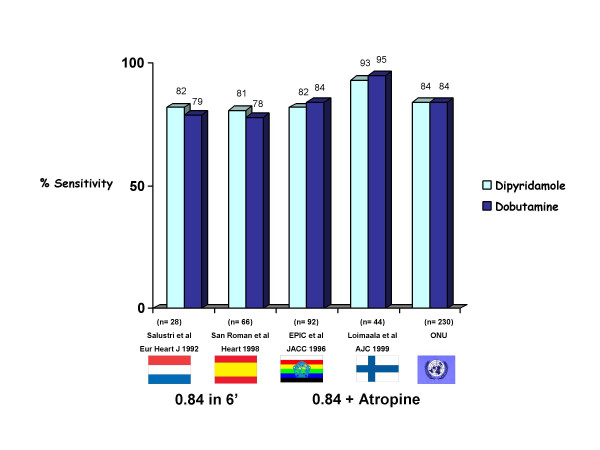
Overall sensitivity of the two most popular pharmacologic stressors, dipyridamole and dobutamine. When the two stressors are used with the same high dose protocol no difference in sensitivity can be found.

**Figure 10:**
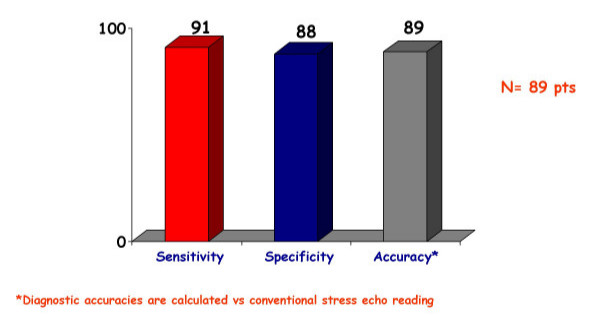
Sensitivity, specificity and diagnostic accuracy of color kinesis employed during stress echocardiography. Please note that the gold standard used to assess accuracies is visual assessment of wall motion abnormalities.

**Figure 11:**
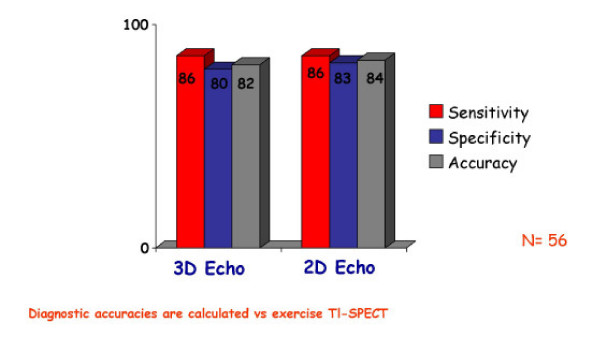
Sensitivity, specificity and diagnostic accuracy of 3D echocardiography vs. 2D echocardiography employed during dobutamine stress echocardiography. No improvement of diagnostic accuracy is observed with the quantitative technique. Please note that the gold standard used to assess accuracies is Thallium-201 SPECT

**Figure 12:**
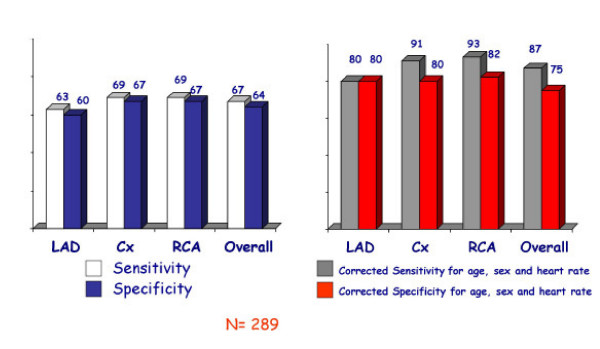
In the left panel sensitivities and specificities in the three main vascular districts and overall sensitivities and specificities without correcting results for age, gender and heart rate. In the right panel, the same data corrected for age, gender and heart rate. No comparison with visual assessment was performed.

**Figure 13:**
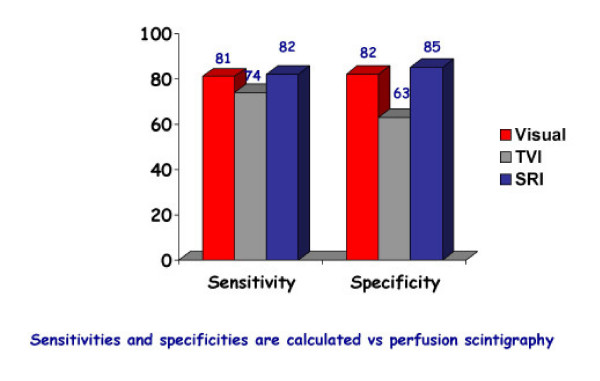
Sensitivity, specificity of tissue velocity imaging (TVI), strain rate and visual assessment during dobutamine stress echocardiography. No improvement of diagnostic accuracy is observed with the quantitative techniques. TVI has an even worse performance than subjective visual assessment. Please note that the gold standard used to assess accuracies is Thallium-201 SPECT

**Figure 14:**
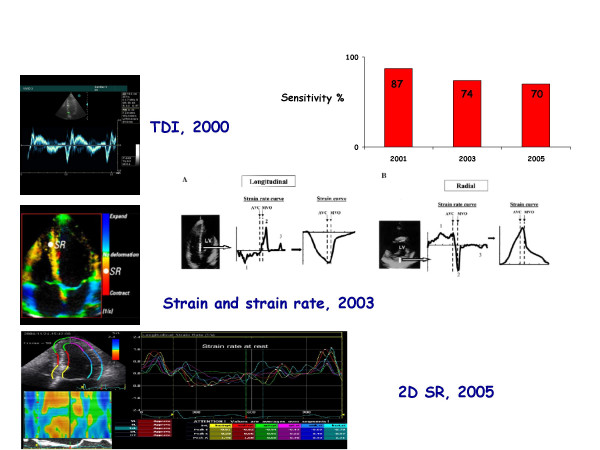
The rise and fall of tissue Doppler (TDI). In a short time lag the technological development has been significant, but for each new implemented Doppler derivative technology the sensitivity of TDI is progressively reduced.

**Figure 15:**
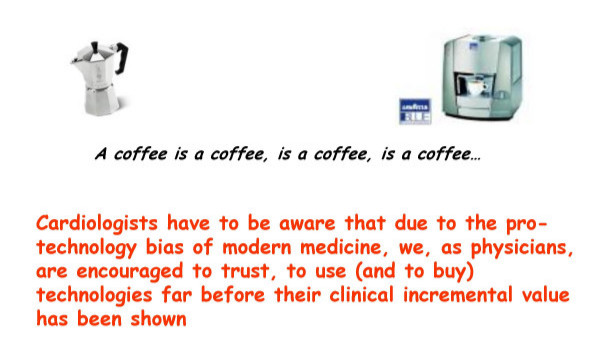
The pro-technology bias. It is a reasonable suspect when data are presented separated by scientific facts. Most of the time expensive technologies produce the same result of older and more established ones.

**Figure 16:**
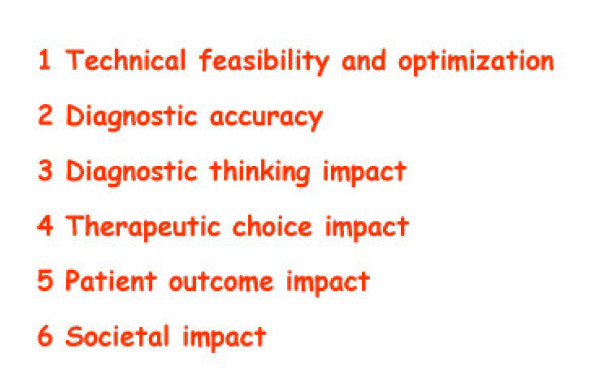
The shape of the technology to come. The Agency for Healthcare Research and Quality Technology Assessment Program has adopted a 6-level framework for evaluating diagnostic technologies. The model emphasizes the need for systematic reviews of diagnostic test studies to go beyond the assessment of technical feasibility and accuracy to examine the impact of the test on health outcomes (12).

**Figure 17:**
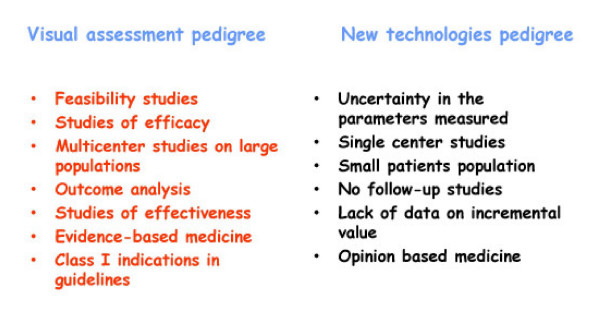
New technologies and stress echocardiography: ready for guidelines? It is necessary to compare newer technologies with the established ones (named Class I indications in the guidelines). Equal is not enough; it has to be demonstrated that they are better.

The EuroEcho9 programme was designed to address some difficult clinical diagnoses in echocardiography such as rare and infiltrative diseases or secondary cardiomyopathies. A special attention was given to the use of echo in intensive care unit and in the surgical setting as a support for continuing monitoring of valve heart surgery. This is my personal evaluation of the best sessions, it is not meant to be objective (I could not follow all sessions), but only to provide suggestions on those areas of research that seemed to be more original : 1 – live 3D echocardiography in particular when used in congenital heart diseases and as a support in interventional pediatric cardiology; it is becoming an important mean in the surgical theatre (Additional file 1) and its employment in cardiac resynchronization therapy seems to have a promising role. 2 – stress echo beyond regional wall motion analysis: new applications for an established technique in the clinical practice. Hence, stress echo for the assessment of valve gradients in aortic stenosis with low ejection fraction or mitral stenosis. The grading of mitral insufficiency in order to assess its dynamic component (Additional file 2 and 3). Stress echocardiography and diastolic function: the hemodynamic consequences of exercise-induced increase in diastolic filling pressure can be demonstrated noninvasively with exercise Doppler echocardiography (Figure 1). The ultrasound lung comets (ULC's) are an echographic sign detectable with cardiac ultrasound on the chest and consisting of multiple comet-tail fanning out from the lung surface (Additional file 4). They may provide diagnostic clues for the assessment and follow-up of patients with pulmonary cardiogenic interstitial thickening. Stress echo and left ventricular contractility: a simple tool to build pressure-volume curves in a non-invasive fashion to assess patients with initial forms of cardiomyopathy or prognosis in those with overt forms of cardiomyopathy. Coronary flow reserve: this new technique has made giant steps in its ability to become an appealing companion to the analysis of wall motion dyssynergy during vasodilator stress echo. Although unable to distinguish between large vessel or microvascular disease, it has become a versatile tool to be employed in different clinical settings: from hypertension with or without left ventricular hypertrophy, to Syndrome X, to athlete's heart, to hypertrophic cardiomyopathy and dilated non-ischemic cardiomyopathy to the follow-up of patients after a successful revascularization. 3 – The "proven clinical" inefficacy of the many technologies sold as breakthrough: color kinesis, tissue characterization, strain rate, tissue Doppler. In a session dedicated to quantitative stress echocardiography and finely chaired by Thomas Marwick and Madler I had the chance to give a talk on the quantitative technologies applied to stress echocardiography entitled "more numbers, more accuracy?". My task was made easier by Thomas Marwick who at the end of each elegant talk dedicated to quantitative methods from color kinesis (Roberto Lang), to 3-dimensional echo (S. Kapetanakis) to contrast (Harald Becher) and tissue Doppler imaging (Rainer Hoffmann), asked the speakers how many times they employed that particular technique in their clinical practice to assess an ambiguous response or on a routine basis: the answers were astonishingly surprising and sincere, a few times or almost never. So nobody argued when the data on quantitative stress echo were presented in comparison with conventional visual assessment with no diagnostic superiority for the quantitative methods at the expense of more time consumed for a single exam and more costs. As we all know, stress echo is a qualitative and operator-dependent technique that with an appropriate training can be performed safely [3,4] and with high diagnostic and prognostic accuracies as demonstrated by large scale multicenter studies performed on thousands of patients [5]. In the face of its strong and sound scientific and clinical pedigree, stress echo, almost every month becomes the technique to be replaced by something better and more accurate and quantitative. The Holy Grail of quantification is a wishful thinking in medicine, since most of the procedures that are employed in the clinical practice are experience and operator dependent: this does not endanger the use of any of them from coronary angiography to calcium score in CT scanning. The conclusion, however, should bring the scientific community to a deep self-criticism: maybe these technologies were sent in the clinical arena without an appropriate evaluation, too soon, too early when they were not ready for a routine clinical use and should have remained inside research laboratories (Figures 2, 3, 4, 5, 6, 7, 8, 9, 10,11, 12, 13, 14, 15, 16, 17) [6-11]

## The Accreditation process

Accreditation in echocardiography has become a very important activity for the Association and the success of the exams, this year also in transesophageal echocardiography in association with European Association of Cardiothoracic Anaesthesiologists (EACTA), is measured by the increasing number of echocardiographists attending the theoretical part. In Florence 31 out of 48 echocardiographists passed the theoretical part of the TTE exam and 37 out 45 of the TEE exam. Kevin Fox and Bogdan Popescu are the masterminds behind this honorable activity. The presence of Luc Mertens inside the Board of EAE as a non-voting member brought clear advantages to the Association such as the first exam in accreditation in congenital heart disease that will be held in Prague, on December 2006, during EuroEcho 10 in association with the European Association for Pediatric Cardiologist (AEPC) and Grown-Up Congenital Heart Disease working group (GUCH). It would be advisable that the National societies and/or working groups recognized the European accreditation exam in order to avoid two parallel processes with different criteria and procedures.

## A proposal for EuroEcho 10

What is missing from the general picture of European echocardiography? The words of Richard Feynman, the physics Nobel Prize, are of help: what is missing is the "perfectly reasonable deviations from the beaten track" [12]. What is the reason for having ten sessions on contrast and no session on contractility? Not to have more pathophysiologic and experimental research performed by ultrasounds? More open discussions on controversial issues that have become standards without passing through scientific validation? [13,14] (Doppler velocities etc. By the way the guidelines on tissue Doppler imaging are under revision).

We should give the echocardiographic scientific and clinical community clear positions and some more "eccentric" (non mainstream) research. Let's keep in mind the warning of Bernard Lown: "Technology in medicine is frequently untested scientifically, often applied without data relating to cost benefit, and driven by market forces rather than by patient needs" [15]. It should be our aim to try to revert this trend.

## Supplementary Material

Additional File 1 Morphological evaluation of a mitral valve by 3D echocardiography. Click here for file

Additional File 2 Apical 4 chamber view of a 70 years old patient with moderate mitral insufficiency in resting condition Click here for file

Additional File 3 and at exercise peak stress (100 w) with the worsening of the grade of mitral insufficiency. Click here for file

Additional File 4 Cardiogenic ultrasound lung comets. Click here for file
